# RELIABLE: Resource Allocation Mechanism for 5G Network using Mobile Edge Computing

**DOI:** 10.3390/s20195449

**Published:** 2020-09-23

**Authors:** Rickson S. Pereira, Douglas D. Lieira, Marco A. C. da Silva, Adinovam H. M. Pimenta, Joahannes B. D. da Costa, Denis Rosário, Leandro Villas, Rodolfo I. Meneguette

**Affiliations:** 1 Computer System Department, São Paulo State University (UNESP), Campus São José do Rio Preto, São Paulo 15054-000, Brazil; rickson.simioni@unesp.br (R.S.P.); douglas.lieira@unesp.br (D.D.L.); 2 Institute of Mathematical and Computer Sciences, University of São Paulo (USP), São Paulo 13566-590, Brazil; marco.colombo@usp.br; 3 Computer Science Department, Federal University of São Carlos (UFSCAR), São Carlos 13565-905, Brazil; adinovam@icmc.usp.br; 4 Institute of Computing, University of Campinas (UNICAMP), Campinas13083-852, Brazil; joahannes@lrc.ic.unicamp.br (J.B.D.d.C.); lvillas@unicamp.br (L.V.); 5 Computer Science Faculty, Federal University of Pará (UFPA), Belém 66075-110, Brazil; denis@ufpa.br

**Keywords:** resource allocation, 5G, MEC

## Abstract

Technological advancement is currently focused on the miniaturization of devices, and integrated circuits allow us to observe the increase in the number of Internet of Things (IoT) devices. Most IoT services and devices require an Internet connection, which needs to provide the minimum processing, storage and networking requirements to best serve a requested service. One of the main goals of 5G networks is to comply with the user’s various Quality of Service (QoS) requirements in different application scenarios. Fifth-generation networks use Network Function Virtualization (NFV) and Mobile Edge Computing (MEC) concepts to achieve these QoS requirements. However, the computational resource allocation mechanisms required by the services are considered very complex. Thus, in this paper, we propose an allocation and management resources mechanism for 5G networks that uses MEC and simple mathematical methods to reduce the model complexity. The mechanism decides to allocate the resource in MEC to meet the requirements requested by the user. The simulation results show that the proposed mechanism provides a larger amount of services, leading to a reduction in the service lock number and as a reduction in the blocking ratio of services due to the accuracy of the approach and its load balancing in the process of resource allocation.

## 1. Introduction

Fifth-generation (5G) networks have been designed to attain a 1000-fold capacity, five-fold reduced latency and 10-fold longer battery life than 4G networks [1,2]. This evolution is due to the proliferation of high-demand mobile applications and the traffic growth exchanged daily by billions of mobile wireless devices worldwide [3]. For instance, a study by Gartner Newsroom stated that around 5.5 million devices were used in 2016, and this number will be approximately 3.8 billion in 2021 [4]. Besides, AT&T states that data traffic on their mobile network has increased by about 250,000% since 2007, and they expected this growth to increase 10-fold by 2020 [5]. This behavior is especially true during the lockdowns implemented due to the COVID-19 concerns—several European Internet Exchange Points (IXPs) reached an all-time peak during this period [6]. Therefore, this rapid traffic growth brings technical challenges in terms of managing mobile devices and meeting different user demands [7].

Fifth-generation networks rely on Mobile Edge Computing (MEC) to provide computational resources at the network edge, reducing latency and improving the Quality of Service (QoS), as MEC reduces communication delay and also data traffic on the back-hall networks [8,9,10]. MEC aggregates idle resources, such as storage and processing, from mobile users sharing the same preferences. Thus, MEC provides Cloud services, such as environment monitoring services, statistical processing calculations, and other services, more closely to mobile users [11,12,13]. In this sense, some mobile users could increase their capabilities by using MEC resources, while other mobile users lend their resources to MEC [14,15]. In MEC scenario, mobile applications and services can be allocated on MEC to meet user demands [16,17]. However, each MEC has a limited resource capacity that can be used to provide services to mobile users [14]. It is therefore essential to design efficient resource management approaches to handle MEC resources to serve a higher number of requested services, minimizing the unnecessary reallocations caused by user mobility.

A resource management mechanism is implemented in two steps: (i) resource aggregation and (ii) resource allocation [18,19,20]. Specifically, the resource aggregation step creates MEC; i.e., a pool of available resources to be used by other mobile devices to meet users’ demands. This involves the announcement of the available idle computational resources, which can be shared with other devices belonging to the system, maximizing the pool of available resources that the system can use. On the other hand, the resource allocation step involves the allocation of services based on the resources available in MEC to better meet the requested services’ requirements. In this context, the choice of when and where the service will be allocated by a resource allocation mechanism greatly impacts the performance of the service provided.

However, the dynamic nature of users leads to a fluctuation in terms of the number of resources in the pool of available resources for a given MEC, where users might frequently disconnect from MEC depending on the user mobility pattern. This issue directly impacts the number of services attended/allocated. Furthermore, user mobility leads to service reallocation inside MEC to maintain service provision. Thus, user mobility highly impacts the service allocation/reallocation performance in terms of service delivery [21]. This is because the service may not be served due to a lack of resources, the service may be blocked in the search for a device to be allocated or reallocated, or it may have its quality of service impaired due to the need to relocate the service. In recent years, many works have proposed different resource allocation mechanisms to allocate services on MEC to provide better services to users [22,23,24,25,26,27,28,29,30]. However, these strategies do not take into account the service length or user mobility information. Besides, such works involve significant complexity in the decision-making process, impacting the computation expense and inference times.

In this article, we propose a resource allocation mechanism for 5G networks, considering Mobile Edge Computing to meet user demand, called RELIABLE—**RE**source al**L**ocat**I**on mech**A**nism for 5G network, considering mo**B**i**L**e **E**dge computing. We consider MEC infrastructure distributed in an urban environment by aggregating idle resources from mobile devices; e.g., vehicles, mobile users and other devices. Thus, each MEC iteration has a limited resource capacity that can be used to provide services to mobile users. In this context, RELIABLE aims to maximize the availability of resources used when requested by MEC. To this end, RELIABLE considers mobility prediction to decrease the unnecessary reallocations caused by user mobility. Service time and network consumption are also used to reduce service allocation and the impacts of reallocation. In this sense, RELIABLE considers a multi-criteria mathematical method to deal with MEC resource allocation to meet the user’s demands for Cloud services. Thus, RELIABLE decides where and when MEC services will be allocated based on the multi-criteria mathematical method. Simulation results show that RELIABLE improved the number of services to be provided compared with allocation mechanisms by 45%; i.e., for Greedy, Best, and Worst mechanisms. RELIABLE also provided a reduction of 40% in the service locks number due to its assertiveness, as well as a reduction in the blocking ratio of services about 45% due to its load balancing in resource allocation. Therefore, the contributions of this work can be summarized as follows:

1. We propose a mechanism to allocate resources in MEC infrastructure as a way to maximize the availability of resources which can be used when they are requested in MEC.
2. We consider the combination of mobility prediction and the resources required, as well as the service time for proper decision making.
3. We perform simulation experiments to introduce the impacts and benefits of RELIABLE, where the results show that RELIABLE can effectively mitigate the challenges related to resource allocation in MEC infrastructure in terms of the number of services served, the number of services blocked and the number of services denied for a different number of users requesting different services.

The remainder of this article is organized as follows. Section 2 describes the most relevant related works in this area. Section 3 introduces the network scenario used in this article, as well as the RELIABLE mechanism and its operation. Section 4 shows the evaluation of the simulation of the performance of RELIABLE and its results. Finally, Section 5 presents the conclusions and directions for future works.

## 2. Related Works

In the literature, some papers have been produced that include MEC resource allocation mechanisms [31,32,33,34,35,36,37,38,39,40]. Song et al. [31] proposed a resource-allocation mechanism based on context-sensitive clustering technology (VNF-RACAG - virtualized network functions resource allocation) to minimize the delay in the service provisioning network. The mechanism uses a stochastic model of Network Function Virtualization (NFV), as well as geographic contexts and user transfer histories in the allocation time optimization process. Furthermore, VNF-RACAG uses individual users’ locations and characteristics to group them into clusters for more efficient functional support. The iterative gradient descent method is used to compute cluster numbers to minimize the end-to-end delay. Next, a graphical partitioning algorithm is used to reduce movement among clusters, which is executed when the user’s trend phase changes. Although the VNF-RACAG mechanism reduces communication time (delay), its complexity increases the processing load of network elements.

van Lingen et al. [32] proposed an architectural approach that addresses some of the technical challenges behind Cloud and Fog communication and resource allocation. This is a model-oriented and service-centric architecture based on OpenFog Consortium (OFC) architecture [41]. This architecture uses a two-layer abstraction model, which provides intercommunication between Cloud and Fog. Additionally, it offers features for specific IoT modules. The approach was applied in Barcelona city, focusing on a small number of use cases. Although this architecture provides intercommunication among all devices, the evaluated environment does not reflect this architecture’s performance in a larger environment with a broader set of IoT devices.

Yu et al. [33] proposed a computational and network resource allocation mechanism for mobile systems using Orthogonal Frequency Division Multiplexing/Multiple Access (OFDMA) as a means of communication. The authors performed sub-carrier allocation for task provisioning and CPU time allocation for task execution in MEC. Thus, OFDMA was proven to be efficient and almost ideal in terms of energy savings for mobile devices in this work. The authors showed that resource allocation significantly impairs system performance through extensive simulations, even though the allocation mechanism for each resource type is almost optimal. Therefore, instead of simply combining the allocation mechanism separately, the two resource types’ congestion information should be considered simultaneously. Moreover, this joint programming approach is more critical when the allocation of computational resources can provide more prominent energy savings.

Ali et al. [36] proposed a resource allocation algorithm based on the device’s power profile using the 5G communication network. This work focused on small devices such as healthcare sensors. The approach is divided into three steps: in the first step, the device’s consumed energy is compared to a selected maximum energy budget value obtained from the QoS metric based on the IoT applications’ power requirements; consequently, the number of sub-carriers is calculated. In the second step, an efficient solution is implemented by inducing a limit value. A given threshold value is selected using mapping based on a QoS metric. The threshold enhances sub-carrier selection for less highly-powered devices. Finally, a suitable threshold value is selected. The threshold value improves sub-carrier calculation for less energy-consuming devices.

Peng and Shen [37] proposed an asset assignment plan to help with distinctive vehicular applications; they considered two normal MEC designs and defined multi-dimensional asset streamlining issues, which as a rule suffered from high calculation unpredictability and an overlong critical thinking time. In this manner, they exploited reinforcement learning (RL) to change the two figured issues and explained them by utilizing Deep Deterministic Policy Gradient (DDPG) and various leveled learning models. Considering vehicles on a two-path straight national street, with one path for every heading, macro eNodeB (MeNB) and Wi-Fi Access Points (APs) were consistently sent to one roadside, with various Wi-Fi APs included in the MeNB approach. Additionally, the authors present the point-by-point range of the board techniques executed by the regulators and introduced by the MeNBand Edge Node (EN)-mounted MEC workers, including range cutting among MeNBs and Wi-Fi APs and range designation among vehicles related to a similar base station (BS).

Agarwal et al. [40] proposed a resource allocation appliance based on VNFs to support the vertical services in 5G networks. They considered that portable system administrators are responsible for planning the prerequisites of the vertical services into a framework of executive choices. This task is a part of the system coordination and involves settling on choices concerning (i) the position of the VNFs required by the verticals over the foundation; (ii) the tasks of CPU, memory and capacity assets for the VNFs; and (iii) the directing of information across organized hubs. These choices are correlated with one another in manners that are perplexing and regularly outlandish. In this paper, the center was around the distribution of computational and system assets, and such choices were considered together, representing (i) the prerequisites of each VNF and vertical service, (ii) the abilities of the system administrator’s foundation and (iii) the limit and inertness of the connections between organized hubs. A key part of this work—regularly ignored by past research on 5G and VNF arrangements—is that our methodology permits the adaptable distribution of the computational abilities of each host among the VNFs it runs.

Wang et al. [38] proposed a resource allocation mechanism based on the VNF choice technique which isolates the vehicular system into a few layers, as indicated by the request for VNFs in an SFC. In each layer, each parcel that appears should be lined first. At that point, the parcel is communicated to a next VNF layer that is chosen by the scheduler. VNFs are chosen by the traffic distinguishing proof, while also considering the system ongoing data transfer capacity and registering assets.

Kiani and Ansari [39] proposed a resource allocation mechanism called Non-Orthogonal Multiple Access - NOMAbased on Edge Computing, which aims to decrease the energy expenditure between the user and the MEC device. To this end, the authors formulated the mechanism to minimize the power consumption of MEC users, optimizing the number of user clusters and the allocation of computing and communication resources, as well as the transmission rate. In particular, similar to frequency resource blocks, the authors split the computing power available in MEC; thus, this approach uses radio-frequency allocation and computational resources for users assigned to different order indices.

Existing resource allocation mechanisms have significant complexity, meaning that MEC can be overwhelmed in the allocation process. Furthermore, most of the existing works do not consider mobility prediction as a decision parameter for carrying out resource allocation. Another important parameter for these mechanisms is the service time; i.e., when the resources will be allocated to attend the requested service, enabling the better management of available resources. Finally, it is essential to consider the computational impact of the execution of the decision mechanism. Depending on the technology used, such as artificial neural networks, the fuzzy approach or other more complex techniques, the execution of the mechanism may affect the execution of the requested services.

Table 1 summarizes the analyzed resource allocation mechanisms, in which we consider user mobility patterns, mobility prediction methods, the service time and the complexity of the method. The method will consume a significant amount of resources which some services could use; for instance, a given work is classified as high if it requires greater overheads and processing in resource management, including methods based on software-defined networks using artificial neural networks. On the other hand, a given work is classified as low if it uses the standard communication protocols and adds an allocation mechanism. Besides, user mobility means the speed at which the mobile user or device moves, where high mobility means vehicles or low mobility means a mobile user or fixed IoT device, for example. Based on our state-of-the-art analysis, we conclude that the works described do not consider the high mobility of the user or the service time and fail to perform a forecast of the mobility of the service flow in which it seeks to reduce unnecessary allocations, given the highly dynamical environment. Therefore, RELIABLE considers all these questions in its decision mechanism with low mathematical complexity. To the best of our knowledge, RELIABLE is the first technique to incorporate all of these critical features in a resource allocation mechanism.

## 3. RELIABLE

In this section, we introduce RELIABLE’s mechanism to maximize the usability of MEC resources available on a 5G network. RELIABLE is designed to handle resource management and allocation for a 5G network to serve a higher number of requested services, minimizing the unnecessary reallocations caused by user mobility. To this end, RELIABLE takes into account the bandwidth, mobility prediction, and service time as input parameters for the allocation decision mechanism. In the following, we introduce the 5G network scenario considering MEC, and we also describe the RELIABLE mechanism in detail.

### 3.1. Network Scenario

We consider a 5G scenario composed of a set of mobile users (e.g., vehicles, mobile users and other devices) $u_{e}$ with an individual identity e∈[1,w], where *w* is the maximum number of users. At any moment, a given mobile user $u_{e}$ could need to run a service, but their computational resources would not support the processing of such a service [14]. In this sense, the device sends a request message to a controller node CN deployed at the 5G network infrastructure to process the service in MEC. We consider MEC $m_{k}$ (k∈[1,o], where *o* is the maximum number of MEC) composed of a group of mobile devices sharing the same preferences that could lend their resources to create a pool of resources that could be made available to 5G network mobile nodes [42]. Specifically, a given mobile node $u_{e}$ might have idle computational resources, such as processing or storage, which can be aggregated and managed by a controller node CN [43].

**Figure 1:**
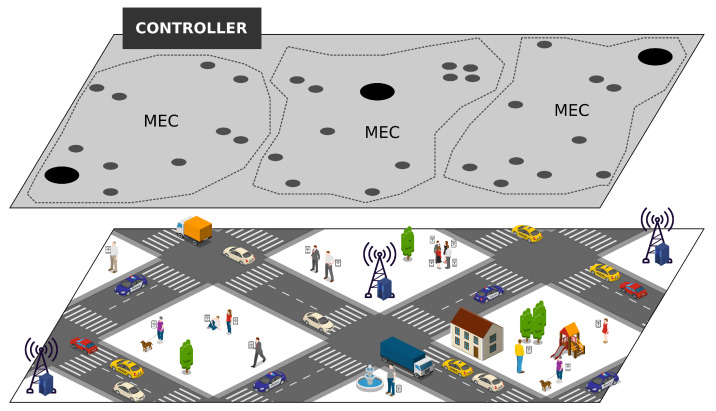
Fifth-generation (5G) network environment considering Mobile Edge Computing (MEC).

In this context, a given mobile node $u_{e}$ could increase its capabilities by using the available resources of MEC $m_{k}$, while other entities lend their resources to MEC $m_{k}$ [44,45]. Therefore, MEC $m_{k}$ could provide services $s_{a}$ (a∈[1,q] where m is the maximum of the number of services) up to $w_{lim}$ mobile users at the network edge. Figure 1 shows the scenario in which the RELIABLE can be deployed in the controller node to manage the resources coming from the urban environment composed of mobile nodes connected through the 5G network infrastructure. The controller node is a centralized entity that has a global view of each MEC iteration and all users to allow better allocation.

In this scenario, a given mobile node $u_{e}$ that may be moving around the urban perimeter may request a given service $s_{a}$—e.g., traffic monitoring or entertainment, among others—from the controller node CN. This service $s_{a}$ requires computational resources, such as processing, storage and runtime, to efficiently serve the user request. In this context, the controller node CN receives the request and takes all decisions regarding when and where to allocate the service $s_{a}$ on a given MEC $m_{k}$ based on the RELIABLE resource allocation mechanism. It is essential to highlight that the controller node CN has an overview of each service and node status to decide which MEC $m_{k}$ has the required resources $r_{serv}$ to allocate a given service $s_{a}$.

Resources made available by MEC $m_{k}$ can be used to allocate a service $s_{a}$ as requested by users. To solve the problem of resource allocation for a given service $s_{a}$, we consider that each MEC can manage up to $q_{serv}$ services, and each MEC supports up to $w_{lim}$ users. Finally, the controller node CN may need to migrate a given service $s_{a}$ to another MEC device to continue to efficiently meet the user’s request. This is due to user mobility and resource availability, or even the need to provide load balance.

### 3.2. Allocation Decision

RELIABLE decides whether or not to allocate resources for a given service $s_{a}$ and which MEC $m_{k}$ such a service will be allocated to in order to meet the needs of the service. To this end, RELIABLE considers an allocation decision step, which takes the decision based on the mobility prediction f(y|x), service time $sev_{time}$ and bandwidth BW.

In this way, the controller node CN monitors the network traffic flows to predict mobility flow between regions; i.e., anticipating the resource utilization in a given place. According to Bui et al. [46], the user network flow follows a continuous distribution in time, and we can use a Gaussian method to predict the user flow among different MECs. This prediction method aims to anticipate the use of resources. For this purpose, Bayesian and the regression of the Gaussian process are used to perform the forecast, and it is necessary to monitor the flows in which they reflect the historical resource allocations used in MEC. Therefore, to carry out this forecasting method efficiently, a particular terminology us defined: the monitoring window, which describes a fixed workload collection period. Table 2 describes the important notations.

Assuming the input data are a collection of locations based on a limited time slot $x=[x_{1},x_{2},x_{3},\ldots ,x_{t}]$, where $x_{t}$ is the location of the flow in the time slot *t*, a finite set of random variables $y=[y_{1},y_{2},y_{3},\ldots ,y_{t}]$ represents the corresponding joint Gaussian distribution of historical flow monitoring statistics regarding the time order. This set, over time, forms the Gaussian process gp, as we can see in Equations (1)–(3), where the prediction function f(y|x) is composed of a kernel function $k(x,x^{\prime })$ [47]. This kernel function is used to define the prior underlying relationship knowledge through a positive–definite function that comprises some special parameters that specify its shape. p(x) is the mean function used to evaluate the time location *x*. Thus, the controller node CN can compute the probability that a given flow from a mobile user $u_{a}$ migrates from one location *x* to another $x^{\prime }$ based on Equation (3).

(1) $$f\left(y|x\right)∼gp(p\left(x\right),k\left(x,x^{\prime }\right))$$

(2) p(x)=E(f(x))

(3) $$k\left(x,x^{\prime }\right)=E\left(\left(f\left(x\right)-p\left(x\right)\right)\left(f\left(x^{\prime }\right)-p\left(x^{\prime }\right)\right)\right)$$

RELIABLE also considers the available bandwidth BW as an input parameter for the selection of the best MEC $m_{k}$ to allocate a given service $s_{a}$. The bandwidth BW is used to check the impact it would have if a given MEC $m_{k}$ allocates the service $s_{a}$; i.e., it evaluates the impact (bandwidth consumption) on the network that the flow exchange would cause, considering that it is necessary to carry out the allocated service. In this way, the controller node CN captures the bandwidth BW, allowing the controller node CN to compute the network impact in case it needs to migrate or allocate a given service $s_{a}$ to another MEC $m_{k}$.

Finally, RELIABLE considers the service length that requires resources to be allocated to fulfill the user request. In this sense, the service time $sev_{time}$ enables us to estimate the start and end time of a given service requested through the sum of the services allocated execution times in MEC $m_{k}$. Thus, the service time $sev_{time}$ is used to compute the duration for which the resources used to serve a given service will be allocated.

RELIABLE calculates different importance degrees for decision making based on the Analytic Hierarchy Process (AHP) method. AHP provides the influence factor for each parameter. It assigns weights to the parameters used; that is, the parameter with the greatest importance over the others will have a greater weight at the end of AHP processing. We consider five importance levels for the comparison between each parameter pair, which indicates the importance of one parameter over the others, as shown in Table 3.

To model the AHP, we consider a weight matrix of $A_{i,z}$, in which the mobility prediction f(y|x) has a higher priority over the other parameters because RELIABLE minimizes the number of unnecessary exchanges caused by user mobility. We also consider that the bandwidth BW has a higher priority over the service time $serv_{time}$ because if the service needs to be transferred, this transfer will not impact the performance of the system. Therefore, the matrix $A_{i,z}$ indicates what influence parameter *i* will have on the other parameters *z*. Table 4 shows the weight assignments used in our AHP. Therefore, these values will be used as a weight to establish the decision matrix, as we can see in Equation (4).

The influence factor $Inf_{i}$ of a given parameter *i* is computed by the sum of the current value multiplication of a metric $F_{i}$; i.e., f(y|x), BW and $sev_{time}$, with the relative importance of the other metrics $A_{i,z}$, as we can see in Equation (4). Therefore, each metric value $F_{i}$ and metric importance $A_{i}$ parameter is calculated by MEC through the applied computational resource monitoring, the service execution time and the bandwidth used.

(4) $$\begin{array}{c} Inf_{i}=F_{i}\cdot \sum_{z=1}^{n}A_{i,z} \end{array}$$

In an urban environment, a given mobile user $u_{a}$ can request different services with different requirements; i.e., a service related to public or driver safety must have a higher priority than services related to advertising a restaurant close to the user. In this way, we divide services into three classes based on [48], where the safety class has priority 1 ($C_{1}$), the comfort class has priority 2 ($C_{2}$) and the entertainment class has priority 3 ($C_{3}$). This priority value for each service is multiplied with the influence factor $Inf_{i}$ to obtain the values of the decision matrix (Equation (5)). The matrix values will be used to choose the best MEC $m_{k}$, in which the resources will be allocated depending on the service importance.

(5) $$\begin{array}{c} M=\left[\begin{array}{ccc} C_{1}\cdot Inf_{1} & C_{2}\cdot Inf_{2} & C_{3}\cdot Inf_{3}\\ \vdots & \vdots & \vdots \\ C_{1}\cdot Inf_{o} & C_{2}\cdot Inf_{o} & C_{3}\cdot Inf_{o} \end{array}\right] \end{array}$$

However, the values of this matrix have a high variation due to the characteristics of each parameter. In this way, we performed a simple normalization of these values to find a more accurate choice, as shown in Equation (6).

(6) $$M_{k,j}=\frac{\left(v_{k,j}-\bar{v_{J}}\right)}{o},$$

where $v_{k,j}$ is the matrix value for MEC K and $\bar{v_{J}}$ is the all-value arithmetic average contained in column *j* of the matrix.

Once data are normalized, we perform a simple calculation of parameter differentiation for each MEC, and the results are stored in a vector as follows.

(7) $$Result=\sum_{j=1}^{n}\left(M_{kj}-F_{k+1,j}\right)$$

where $M_{kj}$ represents the current MEC normalized data and the MEC device to which the resources are allocated, and $F_{k+1,j}$ represents the candidate MEC device’s normalized data to allocate the resource. Therefore, Result contains the values of each MEC device, where the chosen MEC device corresponds to the one with the highest final value (Equation (8)).

(8) $$\begin{array}{c} MEC=Max(Result) \end{array}$$

### 3.3. RELIABLE Operations

Algorithm 1 shows a decision phase overview of the RELIABLE mechanism. RELIABLE must be aware of the available resources at each MEC $m_{k}$, which is provided by communication between MEC devices with the controller node CN, allowing a MEC $m_{k}$ to inform the controller node CN about its idle resources. In this way, these resources become available resource pool parts (lines 1–3). A given MEC $m_{k}$ allocates resources for a given service $s_{j}$, where those resources must be reallocated in another MEC $m_{k^{\prime }}$ before the user $u_{i}$ disconnects from MEC $m_{k}$. It is important to mention that the resource and service migration is beyond the scope of this work, since we focus on the choice of MEC $m_{k}$ to allocate resources for a service $s_{j}$.
**Algorithm 1** Abstraction of RELIABLE1:**if** (MEC_Communication) **then**2: $Pool=Pool+Rec_{MEC\_Resource}$
3:**end if**4:**if** (*request)* **then**5: *probMEC = Func_choose_MEC*6: **while** (probMEC Number_MECs) **do**
7:  **if** ((probMEC_res res_res)&&(probMEC==MECInTraj) **then**
8:   *service_allocated*
9:  **else**
10:   *service_blocked*
11:  **end if**
12: **end while**
13: *service_droped*
14:**end if**

Once a mobile user $u_{i}$ is connected to the controller node CN via the 5G network, it can request a particular service. At each service request, the controller node CN checks which is the best MEC $m_{k}$ to allocate resources to provide such a service. To do this, the controller node CN performs the MEC select function (lines 4–6), which uses AHP and which is also performed when a mobile user $u_{i}$ leaves one MEC device network and connects to another due to mobility. If the selected MEC $m_{k}$ does not have the necessary resources to allocate the service, the next MEC generated by the function will be selected; i.e., the service is blocked until the controller node CN finds a valid MEC $m_{k}$ (lines 7–11). If no MEC $m_{k}$ is selected, the requested service is discarded.

## 4. Performance Analysis

This section describes the performance assessment of the RELIABLE resource allocation mechanism for the 5G network based on a multi-criteria mathematical method.

### 4.1. Scenario Description and Methodology

We implemented RELIABLE’s mechanism using the Python language. We considered that RELIABLE would be executed in an urban environment composed of users moving following a Random Waypoint Mobility model with pause time, as this mobility model enables users to stay in a location in the city (such as a conveniences store) for a while. For RELIABLE evaluation, we considered that the user input and output and MEC followed a Pearson Type III distribution [49].

The simulation considered a variation in the number of users (i.e., 327, 499, 596, 930, and 1088) to represent different situations and to provide a comparison between the best and worst-case (the best case was the simulation with few users while the worst-case was with many users). For the representation of the urban scenario, we considered an area of Manhattan in which the coverage of the urban environment comprised four connected 5G cell towers that allowed interconnection among the MEC devices. Table 5 describes the simulation setup.

We considered two types of services for each MEC device based on the work presented in [48]. Specifically, the security service with the highest priority was prioritized considering the following characteristics: (i) 1 h of service execution time; (ii) 1% of bandwidth consumption for transfer over the 5G network; (iii) 0.5% of processing consumption; and (iv) 1.5% of memory consumption. On the other hand, the second service (i.e., entertainment service) had the following characteristics: (i) 2h of service execution time; (ii) 4% of bandwidth consumption for transfer considering the 5G communication; and (iii) 2.5% of processing consumption; and (iv) 2.5% of memory consumption.

In this way, we evaluated these services over three scenarios to verify the impact of allocating resources from different priority levels of services. Scenario 1 represents the case in which only security services are requested, Scenario 2 shows the case in which only entertainment services are requested, and Scenario 3 shows the case in which the choice of requesting both services is random, and the choice of which service will be requested is also random.

We implemented three resource allocation mechanisms to compare the performance of RELIABLE in the same scenario. Specifically, Greedy runs through the controller until it finds the first MEC device that has the resources it needs. On the other hand, Best runs through the controller by computing the available resource numbers, creating a list of these values and sorting them to choose the most resource-intensive MEC device. Finally, Worst is similar to the previous; it also runs through the controller by computing the available resources numbers, creating a list of these values and sorting them to choose the best MEC device to attempt the service. We can consider that we perform a comparison with the market standard that makes a greedy allocation of available resources. In addition, we compare our method with two other paradigms, in which the Best and Worst scenarios are used to allocate memory. As the existing allocation methods did not consider the prediction of mobility with the time of each service, this would not represent a fair comparison with other methods.

We consider the following metrics to assess the performance of these resource allocation mechanisms:

- The number of services served means the number of services that were allocated in an MEC device.
- The number of services blocked means the number of incorrect choices for service allocation due to the lack of resources available for allocation. Therefore, the service is blocked until RELIABLE finds an MEC device that can allocate the service.
- The number of services denied means the number of requests that, due to lack of resources, were not allocated by any MEC, and thus the number of services that were not actually allocated due to lack of resources by all MEC devices.

### 4.2. Results for Scenario 1

**Figure 2:**
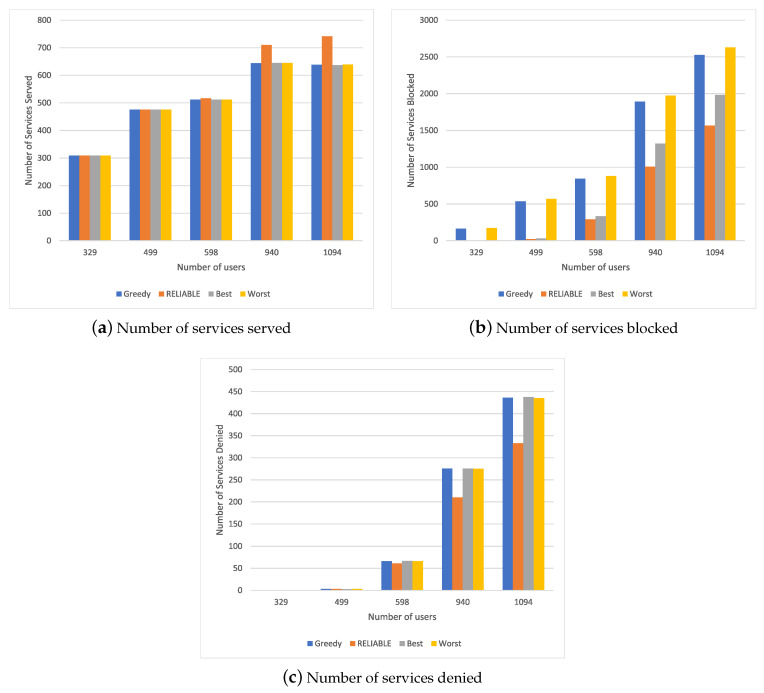
Simulation results for Scenario 1.

Figure 2 shows the simulation results for different resource allocation mechanisms in a scenario with a different number of users requesting the security service. By analyzing the results of Figure 2a, it is possible to conclude that RELIABLE increased in the number of services served compared to other methods, especially in cases with a higher user number requesting the service; i.e., from 940 to 1094 users. This is due to RELIABLE allocating resources based on mobility prediction, as well as the time that the resources would be in execution in each MEC, allowing a better distribution of these resources and the provision of more services. When we analyze a smaller number of users requesting resources, everyone reaches their allocation limit because they can meet all demand without spending a great deal of of time finding an MEC device.

Figure 2b describes the number of times that the service has to wait to be serviced again during a service relocation. We can observe that Best and RELIABLE obtained no blocking since they already selected the best MEC devices to carry out the allocation upon receiving the requests, unlike Greedy and Worst, which had to carry out some reallocations to serve the resources during the service’s execution. When we consider a higher number of service requests, we see that RELIABLE had a better number of blockages, as it avoided an unnecessary transfer of the service, thus obtaining a decrease of 3% compared to Best and 5% compared to the others.

This amount of blocking had an impact on the number of denied services, where RELIABLE obtained a reduction of 2% compared to the other methods, as shown in Figure 2c. When we analyzed a smaller number of requests, we were able to observe that all services were provided with 329 users requesting services, and a very similar services denied number was found 499; this was due to the number of resources available by the set of MEC devices. In this way, when considering the request for only a single service, we can see that RELIABLE can handle a greater number of requests, with a low service blocked number and resource use compared to a scenario with a higher number of requests.

### 4.3. Results for Scenario 2

**Figure 3:**
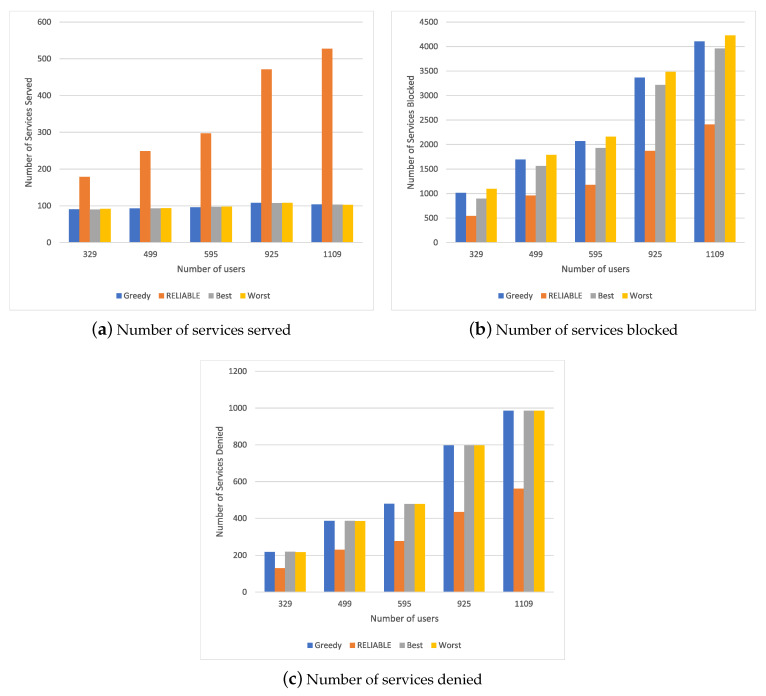
Simulation lresults for Scenario 2.

Figure 3 shows the simulation results for different resource allocation mechanisms in a scenario with a different number of users requesting the entertainment service. Figure 3a shows the number of services served. We can see that RELIABLE could handle more requests than other methods, with a 20% increase considering a smaller number of users and an increase of 38% for a higher number of requests. This is because RELIABLE uses the service execution attributes as well as the user mobility and the resource consumption characteristics of each requested service.

RELIABLE also has a shorter blocking time for switching between one MEC device and another, both for a low and high number of requests, as we can see in Figure 3b. The other methods exhibit similar behavior as these methods provide a greater mobility of services between the MEC devices. The lack of adequate resource balance considerably affects the number of services used by the system, as we can see in Figure 3c. We can observe that the Greedy, Best and Worst methods had the same behavior since their allocation policies did not carry out load balancing among the different MECs, thus overloading only a subset of MECs. Therefore, RELIABLE achieved a 20% reduction in the number of services denied due to an efficient information flow balancing policy.

Based on the results of Scenario 2, we can observe that RELIABLE reduces the number of blocks and meets a greater number of requests, with a low number of blocks and minimal service resources considering the request for only entertainment services, which requires greater computational power and greater bandwidth, as well as a longer service execution time. Comparing Scenario 1 with Scenario 2, we can observe that, with an increase in the resources requested by the service, RELIABLE maintains an increase in the number of services served, with a low amount of denied services. In other words, RELIABLE exhibits stable behavior, as it presented better behavior with an increase in requests, both in terms of meeting service needs with more computational resources and for services that need fewer resources. This is because RELIABLE considers the mobility of the flow and the time that it will be in execution, without using the parameters of the MEC device’s computational capacity in the decision policy. Thus, when we analyze the results obtained in Scenario 1 as well as Scenario 2, both showed an improvement in the increase in users. In Scenario 1 with fewer users (329, 499, 595), RELIABLE showed a behavior similar to the other solution due to the amount of available resources and the low computational quantity required by the service; however, in the transaction and the scenario with a greater number of users (925, 1109), RELIABLE performed better because it balanced its computational load by predicting mobility and the impact allocation or the change of flow. The same can be observed in Scenario 2. Nevertheless, in Scenario 2, it also performed well for a smaller number of users because the amount of resources made available by these users was also greater, which allowed RELIABLE to achieve better management of the available resources.

### 4.4. Results for Scenario 3

**Figure 4:**
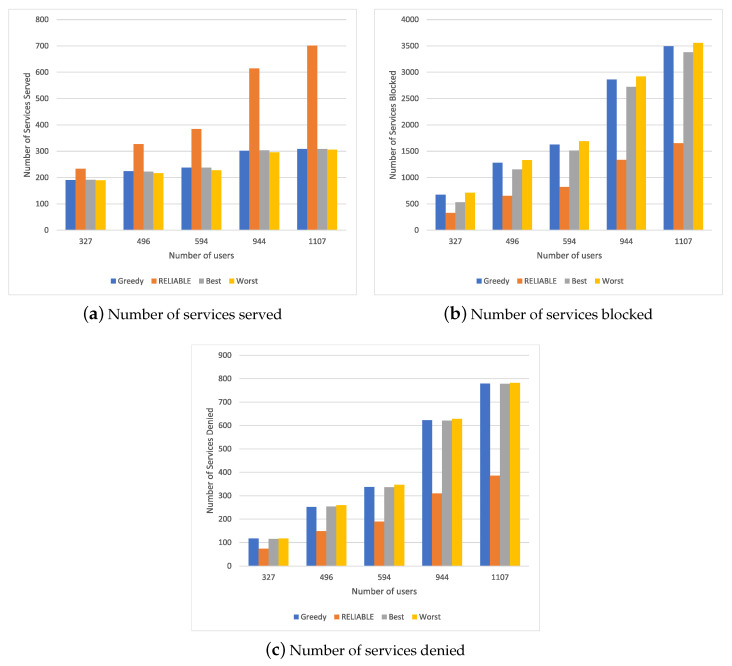
Simulation results for Scenario 3.

Analyzing the results obtained in Scenario 1 and Scenario 2, the behavior of RELIABLE was seen to be focused on attending to one type of service at a time. Thus, it is necessary to check whether this behavior is maintained in a scenario closer to real conditions, in which users could request one or more services at the same time, which requires more or fewer computational resources. Figure 4 shows the simulation results for different resource allocation mechanisms in a scenario with a different number of users requesting both security and entertainment services randomly. RELIABLE achieved a significant increase in the number of services served compared to other methods, as shown in Figure 4a. This is because network parameters regarding service execution are prioritized. Considering a number of users above 580, the Best, Worst and Greedy methods increased the number of services served compared to RELIABLE by 50%. It is possible to see an increase of 5% compared to Random, since it most often selected the MEC devices with sufficient resources to perform the service.

## 5. Conclusions

The service must wait until the resource allocation mechanism finds an MEC device capable of meeting the requirements that the service demands, as shown in Figure 4b. When Best, Worst and Greedy methods are compared to RELIABLE, a reduction close to 47% can be observed. In the case of comparison with Random, the reduction is 7% as the proposed policy selects the first options of MEC devices with sufficient resources to perform the service.

The number of services denied due to a lack of pool resources and Cloud MEC devices can be seen in Figure 4c. When the Best, Worst and Greedy methods are compared to RELIABLE, a reduction close to 39% can be observed. In the case of comparison with Random, the reduction is 3% as the proposed policy has a better load balancing behavior between resources within the MEC; i.e., the Best, Worst and Greedy methods allow the allocation of the available resources in a disorderly manner.

The results show that RELIABLE behaves as expected. RELIABLE remains stable even with different loads of resource requests, and it can therefore serve a higher number of services and consequently block and deny a lower percentage of services. Thus, RELIABLE provides better load balancing in allocations. Analyzing the three scenarios, we can observe that under a mixture of services—that is, a mixture of demands in the network RELIABLE—there was an 18% increase in service attendance for the scenario of higher demand in Scenario 2; however, with a reduction of approximately 1% compared to a scenario with low computational demand. This is because RELIABLE manages to prioritize which services will be allocated and where resources will be allocated to meet such services. When we consider the number of blocked and the number of denied services, we could observe a reduction of approximately 13% of blocked services and 10% of denied services compared to Scenario 2 and a small increase of approximately 27% of blocked services and 25% compared to Scenario 1. This may lead to a greater demand for services close to Scenario 2. However, RELIABLE performed better than the other comparison mechanisms—Greedy, Best and Worst.

In this article, we proposed RELIABLE, which addresses the resource allocation problem in 5G networks using MEC. We consider an MEC network composed of a set of mobile devices which have available resources to be shared, increasing the number of services offered by the MEC network. To this end, we designed a multi-criteria decision-making method and AHP that considered not only the service and network parameters but also the mobility of the flow. Therefore, the decision-making method offers a balanced input with different degrees of importance, maximizing resource utilization in the Cloud. The numerical results show that RELIABLE allows a larger amount of services to be provided, providing a reduction in the number of service blocks due to its assertiveness, as well as a reduction in the number of services denied due to its load balancing in resource allocation. In future work, we will consider other parameters, such as energy consumption and mobility, to improve the mechanism.

## Figures and Tables

**Table 1:** Summary of resource allocation mechanisms.

Works	User Mobility	Mobility Prediction	Service Time	Method Complexity
Song et al. [31]	high	no	no	high
Lingen et al. [32]	low	no	no	high
Yu et al. [33]	low	no	no	low
Ali et al. [36]	low	no	no	low
Peng and Shen [37]	low	no	no	high
Agarwal et al. [40]	low	no	no	high
Wang et al. [38]	low	no	no	high
Kiani et al. [39]	low	no	no	high
**RELIABLE**	**high**	**yes**	**yes**	**low**

**Table 2:** List of important notations.

Term	Description
$u_{e}$	Mobile node (user devices)
*w*	Maximum number of mobile nodes
$w_{lim}$	Maximum limit of connected users at an edge
$m_{k}$	Available resources in MEC k
*o*	Maximum number of MEC
$s_{a}$	Service α
*q*	Maximum number of services available from MEC
$q_{service}$	Maximum service managed by MEC
$r_{serv}$	Required resources
f(y|x)	Mobility prediction
$sev_{time}$	Service time
BW	Bandwidth
CN	Controller node
*x*	Vector of location based on time slot
$x_{t}$	Location of the flow in the time slot *t*
*t*	Maximum number of time slots
*y*	Joint Gaussian distribution of historical (random value)
gp	Gaussian process
$k(x,x^{\prime })$	Kernel function
p(x)	Mean function to evaluate at the time location
$A_{i,z}$	Decision matrix
*M*	Normalized matrix

**Table 3:** Pairwise importance levels.

Value	Degrees of Importance
3	The parameter is much more important than the others
2	The parameter is more important than another
1	Two parameters have the same importance
1/2	The parameter is less important than another
1/3	The parameter is much less important than the others

**Table 4:** Influence factor. BW: bandwidth.

Factor	f(y|x)	BW	${\mathrm{sev}}_{\mathrm{time}}$
f(y|x)	1	2	3
BW	1/2	1	3
$sev_{time}$	1/3	1/3	1

**Table 5:** Simulation setup.

Parameter	Value
Maps	Manhattan city
Number of users	327, 499, 596, 930 and 1088
Cellular Network	Four connected 5G cell towers
User input and output in MEC	Pearson Type III distribution
Services	Security and entertainment
Security service time	1 h
Security service bandwidth consumption	1%
Security service memory consumption	0.5%
Security service processing consumption	1.5%
Entertainment service time	2 h
Entertainment service bandwidth consumption	4%
Entertainment service memory consumption	2.5%
Entertainment service processing consumption	2.5%
